# Freshwater ecotoxicity characterization factors for PFASs

**DOI:** 10.1093/inteam/vjae013

**Published:** 2025-01-06

**Authors:** Rahul Aggarwal

**Affiliations:** Environmental Systems Analysis, Chalmers University of Technology, Gothenburg, Sweden

**Keywords:** life cycle assessments, USEtox, characterization factor, ecotoxicological effect factor, PFAS

## Abstract

This research aims to address the data gaps in freshwater ecotoxicological characterization factors (CFs) for per- and polyfluoroalkyl substances (PFASs). These CFs are essential for incorporating the ecotoxicity impacts of PFAS emissions into life cycle assessments (LCAs). This study has three primary objectives: first, to calculate a comprehensive set of experimental aquatic ecotoxicity CFs for PFASs utilizing the USEtox model (version 2.13); second, to compare these newly derived CFs with those generated using the PFAS-adapted USEtox model; and finally, to test the hypothesis concerning a potential correlation between CFs and effect factors (EFs) with the number of perfluorinated carbons in PFASs. In this study, 367 PFASs were selected from the CompTox Chemicals Dashboard PFAS suspect lists and REACH (Registration, Evaluation, Authorisation and Restriction of Chemicals) registration dossiers. Experimental ecotoxicity data were extracted from CompTox Version 2.1.1 and REACH. Using both the USEtox model (version 2.13) and the PFAS-adapted USEtox model, CFs were calculated for 367 PFASs. Of these, 237 CFs were newly calculated using the HC20_EC10eq_-based methodology, enriching the representation of PFASs in LCA studies. The analysis revealed no correlation between the number of perfluorinated carbons and the calculated EFs and CFs using the USEtox models. This study covers only a small portion of the extensive list of millions of PFASs in PubChem, primarily due to data constraints and scarcity. Discrepancies between CFs generated by USEtox and PFAS-adapted USEtox are attributed to variations in foundational fate and exposure factor calculation methodologies, whereas ecotoxicity factors remained consistent. Consequently, it is suggested that CFs for PFASs are dependent on the modeling approach and require regular updates with the latest data to ensure accuracy and relevance.

## Teaser

**Key points**
 

- This study addresses data gaps in freshwater ecotoxicological characterization factors (CFs) for PFAS substances in life cycle assessments (LCAs)
- This study calculates and compares CFs for 367 PFASs using both the USEtox model (version 2.13) and the PFAS-adapted USEtox model
- This study finds no correlation between CFs or effect factors (EFs) and the number of perfluorinated carbons in PFAS substances
- This study highlights the lack of ecotoxicity data for PFAS chemicals, which hinders the calculation of characterization factors (CFs) for these substances

## Introduction

Over the past decades, the use of polyfluoroalkyl substances (PFASs) in consumer products has surged alongside growing global consumption, making these chemicals integral to nearly all manufactured goods (Bălan et al., 2021; Dewapriya et al., 2023; Glüge et al., 2020; Ng et al., 2021; UNEP, 2019). The world's population, now exceeding 8 billion, owes much of its growth and progress to chemical innovations including PFASs, affecting sectors such as energy, transportation, and agriculture (Barrett, 2000; Ogunseitan, 2023; Perera & Meegoda, 2024; UNEP, 2019). This prolific chemical landscape is evident in the Chemical Abstracts Service (CAS) cataloging of over 200 million entities since the 1800s, including millions of PFASs and approximately 350,000 commercially registered chemicals (CAS, 2023; ECHA, 2023b; Schymanski et al., 2023; Wang et al., 2020). However, this advancement comes at a cost, as chemicals have led to toxic pollution, adverse health effects, and ecological damage (Abunada et al., 2020; Ankley et al., 2021; Donley et al., 2024; Hekster et al., 2003; Panieri et al., 2022). In the planetary boundary concept, chemical pollution was one of the original nine planetary boundaries but was later renamed the “novel entities” (NEs) boundary and remains inadequately understood, posing risks to human sustainability and exacerbating other global challenges like climate change and biosphere integrity (Cousins et al., 2022; Diamond et al., 2015; Rockström et al., 2009; Steffen et al., 2015; Wang et al., 2020). Cousins et al. (2022) argue that NEs, rather than being a single planetary boundary, can be seen as a placeholder for multiple boundaries related to NEs, with PFASs defining a new planetary boundary within this category.

Chemicals classified as PFASs have raised significant environmental and health concerns (Gerald T Ankley et al., 2021; Cousins et al., 2020; Kwiatkowski et al., 2020). Per- and polyfluoroalkyl substances are in use in many applications and products, including fire-fighting foam, electroplating, ammunition, climbing ropes, artificial turf, soil remediation, and many other applications (Aminot et al., 2023; Glüge et al., 2020). The European Chemicals Agency (ECHA) defines PFASs as “substances containing at least one aliphatic CF2 or CF3 element” (ECHA, 2023a; Wollin et al., 2023), such as polytetrafluoroethylene, known as Teflon (Herzke et al., 2012) and perfluorooctane sulfonate (Wee & Aris, 2023). Research studies including Arp and Hale (2019), Hale et al. (2020a), and Neumann and Schliebner (2017) have stressed the importance of ensuring that the REACH (Registration, Evaluation, Authorisation and Restriction of Chemicals) registration process acknowledges the risks associated with PFASs. In 2020, the European Commission unveiled plans as part of its Chemical Strategy for Sustainability to phase out most PFAS applications, and in 2023, ECHA published a proposal under REACH to restrict PFAS production, use, and commercial distribution, including imports (EU, 2020; Wollin et al., 2023). PFASs have been identified as contaminants in drinking water sources (Abunada et al., 2020; Hale et al., 2020b). A significant concern lies in their ability to evade traditional wastewater and drinking water treatment processes (Amen et al., 2023; Banks et al., 2020; Garg et al., 2021; Van Der Hoek et al., 2014; Xiao et al., 2023).

In past decades, life cycle assessment (LCA) methodology, with available standards such as ISO 14040, has gained recognition for its ability to quantify potential toxicity impacts across a product's life cycle (Arvanitoyannis, 2008; ISO, 2006a, 2006b; Rosenbaum, 2015; Tickner et al., 2021). Although existing LCA methods allow for the theoretical assessment of potential toxicity impacts associated with products and processes containing PFASs, the practical incorporation of these chemical emissions into toxicity impact assessment relies on the availability of characterization factors (CFs) for each chemical emission (Aggarwal, Holmquist, et al., 2024; Aggarwal & Peters, 2024; Holmquist et al., 2020; Roos et al., 2018). These CFs serve as a vital link between chemical emissions and potential toxicity impacts within an LCA (Pennington et al., 2004; Rosenbaum et al., 2008). Despite the potential ecological risks posed by PFAS chemicals, only a limited number have undergone characterization for LCA, thereby leading to data gaps and noninclusion of these emissions in assessments (Aggarwal, Holmquist, et al., 2024; Aggarwal & Peters, 2024). In the context of the PFASs and other fluorinated compounds listed in PubChem under the CompTox Chemicals Dashboard PFAS suspect list, a staggering 16,120 PFASs are identified (CompTox, 2023; PubChem, 2023). In contrast, the USEtox database (version 2.01) accounts for just 14 of these PFASs from the suspect list (Fantke et al., 2017). Consequently, there exists a significant gap in available CFs related to PFASs, impeding their integration into LCA methodologies. For the calculation of the CFs associated with freshwater ecotoxicity impacts, fate-exposure-effect models such as USEtox are commonly used (Fantke et al., 2017; Hauschild et al., 2008; Rosenbaum et al., 2008). USEtox is the UNEP/SETAC scientific consensus model for the calculation of the CFs for human toxicity (cancer and noncancer) and ecotoxicity (freshwater) (Fantke et al., 2017).

In recent years, as part of the European Green Deal (Fetting, 2020) aimed at achieving zero pollution, the product environmental footprint methodology was developed to evaluate the potential impacts of chemicals within the LCA framework alongside other impact categories (Abbate et al., 2024; Damiani et al., 2022). The USEtox consortium and the European Commission are continuously researching and updating the methodology for calculating CFs and adding more chemicals to the CF database (Owsianiak et al., 2023; Sala et al., 2022). These research-related updates, along with additional literature, have reduced limitations in including chemical emissions within the product environmental footprint (Saouter, Biganzoli, et al., 2019; Saouter, Wolff, et al., 2019). Particularly, Saouter et al. (2018) calculated freshwater ecotoxicity CFs for chemicals in an EU publication using substance hazard values derived from the 20% effect value from species sensitivity distribution, based on chronic EC10 equivalent aquatic toxicity data. These data were collected from REACH, the European Food Safety Agency (EFSA), and the Pesticide Property Database (PPDB), and incorporated into an updated USEtox method. This resulted in HC20 values with data for at least three trophic levels for 6,764 chemicals from REACH/EFSA and for 1,316 chemicals from PPDB. The calculation of CFs using the HC20_EC10eq_ based approach instead of the HC50_EC50eq_ based approach was recommended in the methodology proposed by Owsianiak et al. (2023).

This study addresses the substantial knowledge gap arising from the absence of aquatic ecotoxicological CFs for PFASs listed in the CompTox Chemicals Dashboard PFAS suspect list and REACH registration dossiers. The study has three objectives: first, to calculate a comprehensive set of experimental aquatic ecotoxicity CFs for PFASs using the USEtox model (version 2.13); second, to compare these newly derived CFs with those generated using the PFAS-adapted USEtox model; and third, to investigate a potential correlation between CFs and EFs with the number of perfluorinated carbons present in PFASs.

## Methods

### PFAS substance selection

This research begins by assembling a group of compounds categorized as PFASs. Given the evolving nature of PFAS assessment criteria and its definition, the study relies on existing literature regarding these compounds (Brennan et al., 2021; Gaines et al., 2023; Kwiatkowski et al., 2020). Instead of categorizing substances directly as PFASs, the primary objective of this study was to assemble a list of chemicals that are either officially acknowledged or have the potential for PFAS classification from PubChem (PubChem, 2023). Therefore, the emphasis is on assembling this list from openly accessible sources that adhere to well-defined and transparent PFAS classification and inclusion criteria.

### Characterization factor calculation tool

Evaluation of the ecotoxicity impact of a chemical requires the calculation of its environmental fate, exposure, and ecotoxicological effects (Jolliet et al., 2006). The USEtox model provides a systematic framework for calculating ecotoxicity CFs of chemicals (Fantke et al., 2017; Hauschild et al., 2008; Rosenbaum et al., 2008). In the USEtox modeling, ecotoxicity CFs [Potentially affected fraction of species (PAF)‧m^3^‧day/kg-emitted] are determined by the matrix computation of three factors: fate factors (FF, kg‧kg^−1^‧day^−1^), environmental exposure factors (XF), and freshwater ecotoxicological effect factors (EF, PAF‧m^3^‧kg^−1^) (Fantke et al., 2017; Owsianiak et al., 2023) using the matrix equation: CF = FF × XF × EF, where FF represents the substance residence time in a freshwater compartment for a given unit of time, XF represents the dissolved fraction of the substance in the freshwater compartment, and EF represents the relationship between the potentially affected fraction (PAF) of aquatic species and the concentration of a substance, derived from species sensitivity distribution (Rosenbaum et al., 2008, 2007).

In this study, four methodologies for the calculation of the CFs were used. These methodologies differ in the calculation of the EFs, FFs, and XFs. Starting with the first methodology, the USEtox model (version 2.13) was used to compute aquatic CFs for the identified PFAS chemicals. This is the official methodology of USEtox, where EFs correspond to the 0.5 PAF in the linear concentration-response relationship of the HC50 [kg m^−3^] (Fantke et al., 2017). The equation used is EF = 0.5/HC50_EC50eq_ [PAF m^3^ kg^−1^]. The HC50 is the concentration at which 50% of species in a freshwater ecosystem are exposed to levels exceeding their EC50, calculated from the geometric mean of chronic EC50 values for different freshwater species. The EC50, on the other hand, refers to the concentration at which half of the exposed test species population experiences a measurable effect. This approach resulted in HC50_EC50eq_ based on chronic EC50 values for individual chemicals, which were then used for the calculation of the EFs.

The second methodology is based on the recommendations proposed by Owsianiak et al. (2023) for the calculation of the EFs using the HC20_EC10eq_ based approach. HC20 is defined as the environmental concentration of a chemical affecting 20% of aquatic species. The effect on individual species is determined using EC10 chronic equivalent ecotoxicity data for the aquatic species, represented by the equation EF = 0.2/HC20_EC10eq_ [PAF m^3^ kg^−1^] (Sala et al., 2022). This approach resulted in EFs based on HC20_EC10eq_ for individual chemicals, which were then used for the calculation of the CFs, thereby aligning with the latest guidelines for calculating EFs.

The third methodology applied in this study acknowledges that USEtox is not specifically tailored to account for the unique characteristics of PFAS chemicals, as it serves as a general global consensus tool for various chemicals (Holmquist et al., 2020). Owsianiak et al. (2023) identified key challenges in characterizing chemical substances classified as PFASs within their proposed recommendations as given in second methodology. These challenges include considering the degradation of PFAS compounds into potentially toxic degradation products and accounting for the unique chemical characteristics of PFAS, such as their combination of lipophobic and hydrophobic properties. These properties make conventional Kow-based partitioning models, typically used for other organic substances in USEtox model (version 2.13), inapplicable. To address these issues, a PFAS-adapted model was developed by Holmquist et al. (2020) based on an earlier version of USEtox (version 2.1). This PFAS-adapted model introduced several key enhancements to better suit the assessment of PFAS ecotoxicity impacts and using Koc instead of Kow for chemical partitioning. In this study, a PFAS-adapted model was used, but not the one from Holmquist et al. (2020), which was based on an earlier version of USEtox (version 2.1). Instead, the USEtox model (version 2.13) was adapted, following the same recommendations as Holmquist et al. (2020) to calculate the CFs. However, species richness was not included in the EF calculation to maintain consistency with the available EF calculation methods in USEtox (version 2.13), ensuring uniformity between the two models for comparison. This resulted in the EFs based on HC50_EC50eq_ for individual chemicals for the calculation of the CFs.

The last methodology is similar to the third methodology, with the key difference being that instead of EFs based on HC50_EC50eq_ for individual chemicals, the PFAS-adapted model uses EFs based on HC20_EC10eq_ for the calculation of the CFs. This approach incorporates the specific characteristics of PFASs and aligns the EFs with the latest guidelines, ensuring that the unique environmental behavior and toxicity profiles of PFAS chemicals are accurately represented.

### Data collection for PFASs

In the calculation of ecotoxicity CFs using USEtox, a set of essential input data parameters is required (Fantke et al., 2017). This study compiled a comprehensive dataset of physical-chemical properties sourced from three key references: CompTox, EPI Suite v4.11, and U.S. Environmental Protection Agency (USEPA) TEST version 5.1.2 (USEPA, 2020, 2023b; Williams et al., 2017). Data collection strategy involved extracting information with a primary focus on acquiring openly accessible experimental data whenever possible. In cases where experimental data were not available, this study collected estimated physicochemical data from QSARs (quantitative structure activity relationships). To ensure data accuracy, this study removed duplicate entries and computed averages for datasets containing multiple data points. The dataset was categorized into two sets: experimental and estimated, and all units were standardized to align with USEtox specifications. The data priority sequence of this study placed experimental data first, followed by estimated data. In instances where data were entirely absent, conservative assumptions, such as chemicals being neutral for parameters like pKaChemClass, pKa.gain, and pKa.loss, were made. This approach ensured consistent integration of experimental data into the USEtox framework for CF calculations, but in their absence, harmonized estimated data were utilized. This is based on the previous work by Aggarwal and Peters (2024).

To compile a robust dataset of aquatic ecotoxicity data, this study curated data from two open-access platforms with the primary source being experimental ecotoxicity data from the REACH databases (REACH, 2020) complemented by the CompTox version 2.2.1 database (Adkins, 2023; Williams et al., 2017). Information from the REACH databases was retrieved in August 2020, and data from the CompTox database, which aggregates information from ToxValDB v9.4, were acquired in July 2023. The REACH database provided 1,274 toxicity raw data points for 88 chemicals identified by their CAS numbers. The CompTox database provided 30,480 ecotoxicity raw data points for 315 chemicals.

### Harmonization of ecotoxicity data

In this study, a data harmonization process consisting of four key steps was used to standardize the collected experimental ecotoxicological data for PFAS building on the previous work by Aggarwal and Peters (2024) and Aggarwal (2024). The first step, chemical identification, was vital for associating each PFAS data entry with its specific chemical entity. CAS numbers were designated as the primary identifiers. PFAS entities lacking CAS identifiers were excluded from the harmonization process. In the second step, data quality assessment played a critical role in ensuring data reliability. For the ECHA dataset, data reliability was assessed using Klimisch scores, with scores other than 1 or 2 signifying unreliability and leading to data exclusion (Klimisch et al., 1997). In the CompTox data set, data trustworthiness was determined by the QC status, with only entries marked as “pass” progressing to further analysis.

The third step of this study's data harmonization process focused on achieving uniformity across various key parameters. Numeric qualifiers other than “=” were eliminated to ensure precision. Species tested were categorized using the common species names from the USEPA ECOTOX knowledgebase (USEPA, 2023a), and they were assigned to predefined species groups, with any species not fitting these categories being removed. Exposure durations were standardized to days and assigned acute and chronic exposures, taking into account chronic exposure thresholds as outlined by Aurisano et al. (2019). Endpoints were consolidated into two primary categories: EC10eq, and EC50eq, denoted as EC10 and EC50 for simplicity. Furthermore, effect units were standardized to milligrams per liter (mg/L), and data points with missing effect values or unclear units were systematically excluded. This process ensured a consistent, reliable, and high-quality dataset for subsequent analyses. Finally, in the last step, a consistency check was executed to ensure that the collected data was purely experimental, complete, and devoid of duplicates and redundancies.

This study has limitations related to the harmonization and collection of data. The experimental data used to calculate the CFs were gathered from the REACH and the CompTox databases. However, not all study-related information for each data point is consistently available in these databases. As a result, some experimental considerations were not fully accounted for, including the chemical purity of the ecotoxicity tests, differences in experimental design (e.g., flow-through systems vs static tests), the distinction between guideline studies and novel designs, and the selection of the most relevant and sensitive endpoint per study, per chemical, per test species, and per exposure level.

### Comparing effect and characterization factors

The comparison of ecotoxicity EFs and CFs calculated with four methodologies described in the “Characterization factor calculation tool” section, along with available USEtox CFs for 58 overlapping chemicals from the USEtox organic substances database (version 2.01) serves as the focus of the analysis. To investigate these relationships, this study first applied a log10 transformation and then performed pairwise correlation analysis. The resulting correlation coefficient (r) represents both the direction and strength of these linear relationships. To delve further into the robustness of these correlations, this study calculated coefficients of determination (R^2^) of the regression analysis. This study also analysis other available studies to compare PFAS CFs. One of these studies, conducted by Saouter et al. (2018), calculated CFs for 6,711 chemicals. However, the overlap with PFAS chemicals in this study is 130 chemicals with ecotoxicity CFs for emissions to the freshwater compartment. In Saouter et al. (2018), CFs are based on HC20_EC10eq_ using the USEtox 2.1 model. In 2023, the model was updated to USEtox version 2.13, which includes corrections related to land area fractions for continental and subcontinental scales (USEtox, 2023). In this study, the results from Saouter et al. (2018) are denoted by the JRC Technical Report, as it is part of the technical report by the Joint Research Centre, the European Commission’s science and knowledge service. Other studies that include PFAS CFs based on HC50_EC50eq_ include a study by Aggarwal and Peters (2024), which has 18 overlapping chemicals, and a study by Aggarwal, Holmquist, et al. (2024), which has 20 overlapping chemicals.

### Group analysis between CFs and number of perfluorinated carbons

In this study, a simplified group analysis was developed relating the number of perfluorinated carbons (CF, CF2, and CF3 functional groups), with the calculated EFs and CFs. This is an attempt to understand whether there is any correlation pattern between these that can be used to fill gaps in the absence of the required data to calculate EFs and CFs for PFASs. However, it is important to note that other parameters instead of chain length could also be used for such correlations, such as molecular descriptors in machine learning techniques (Hamid et al., 2023; Lai et al., 2022; Meng et al., 2024).

## Results and discussion

### PFASs selected

The study began by compiling a list of chemicals previously identified or suspected as PFASs, rather than categorizing new chemicals as PFASs. This list was curated from transparent sources. An initial literature review led to the PFAS and Fluorinated Compounds list in PubChem, which appeared to be a comprehensive source of PFASs (PubChem, 2023). The cornerstone of this study was the CompTox Chemicals Dashboard PFAS suspect list and the REACH registration dossiers (CompTox, 2023). Based on the Organisation for Economic Co-operation and Development (OECD) 2021 definition for PFASs, which defines them as chemicals containing at least one saturated CF2 or CF3 part, a total of 367 chemicals were selected from the available PFAS list, considering the availability of data for the calculation of the CFs (Chelcea et al., 2020; OECD, 2021; Wang et al., 2021).

### Ecotoxicity data selection and harmonization results

This research incorporated experimental ecotoxicity data from two primary sources, REACH and CompTox, to construct a harmonized dataset for CFs computations as provided by Aggarwal (2024). The raw data underwent a four-step harmonization process, elaborated in the “Harmonization of ecotoxicity data” section. Post harmonization, this resulted in 22,553 ecotoxicity data points associated with 367 PFAS chemicals. The data primarily include species groups, with 51.0% of the data corresponding to fish, 16.6% to crustaceans, 10.7% to algae, 5.9% to insects/spiders, 5.3% to aquatic plants, 4.3% to mollusks, and 1.4% to invertebrates, among others. All the information regarding the number of ecotoxicity data points per chemical, the corresponding number of species, and the number of species groups is available in the online supplementary material Table S1. The distribution of the endpoints is shown in Table 1.

**Table 1.** Overview of the distribution of harmonized ecotoxicity datapoints across different endpoints.

Type	EC10 acute	EC10 chronic	EC50 acute	EC50 chronic	NOEC acute	NOEC chronic	Total
**Datapoints**	2795	3653	5833	1444	3693	5135	22553
**Chemicals**	157	178	254	161	222	226	367

*Note:* EC = Effect concentration; NOEC = No Observed Effect Concentration.

### Ecotoxicity effect factors calculation results

In this study, EFs were derived for a total of 367 PFAS chemicals using first, methodologies 1 and 3 with experimental EC50 chronic data and, when necessary, endpoints extrapolated to EC50 chronic using the extrapolation factors provided in Aggarwal, Gustavsson, et al. (2024). Second, for methodologies 2 and 4, using experimental EC10 chronic data and, when necessary, endpoints extrapolated to EC10 chronic. Further details on the summary statistics of EFs are available in the online supplementary material Table S1.

**Figure 1.**
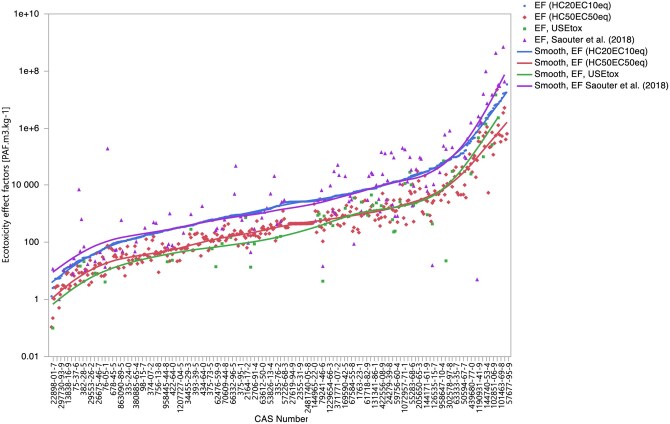
Calculated ecotoxicity effect factors (EFs) for 367 polyfluoroalkyl substances based on HC20_EC10eq_ and HC50_EC50eq_, including overlaps with the USEtox database (version 2.01) for 58 chemicals (based on HC50_EC50eq_) and overlaps with Saouter et al. (2018) for 130 chemicals (based on HC20_EC10eq_). The *x* axis is arranged in ascending order of EF values based on the HC20_EC10eq_ approach. *Note*: HC20_EC10eq_ = Hazardous concentration affecting 20% of aquatic species based on EC10 chronic equivalent data for the aquatic species; HC50_EC50eq_ = Hazardous concentration affecting 50% of aquatic species based on EC50 chronic equivalent data for the aquatic species.

The calculated EFs exhibit a range. To visually illustrate this variation, Figure 1 presents EFs based on the two calculation methods used. Also, the 58 PFAS chemicals already present in the USEtox database and 130 PFAS chemicals from Saouter et al. (2018) are also shown. These graphical representations effectively showcase the distribution of EFs, highlighting the range of values.

### Comparison of ecotoxicity effect factors

**Figure 2.**
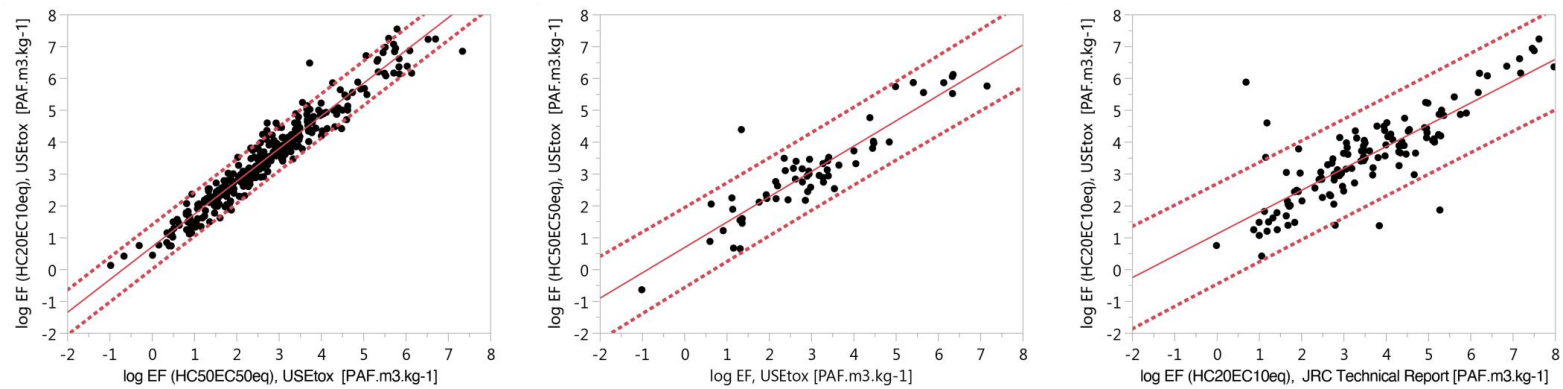
Regression analysis of log-transformed effect factors (EFs) [Potentially affected fraction of species (PAF) m³ kg^−1^] based on HC20_EC10eq_ in the freshwater ecosystem versus log-transformed EFs [PAF m³ kg^−1^] based on HC50_EC50eq_ calculated in this study with experimental ecotoxicity data (left), with EFs from the USEtox database (middle), and with EFs from Saouter et al. (2018) (right). The correlations are as follows: very strong (*n* = 367, R^2 ^= 0.94, r = 0.97), strong (*n* = 58, R^2 ^= 0.83, r = 0.91), and moderate (*n* = 130, R^2 ^= 0.70, r = 0.84) respectively.

The comparison of EFs focused on EFs derived from the experimental data in this study, based on HC20_EC10eq_, with the calculated EFs based on HC50_EC50eq_, as illustrated in Figure 2 and Table 2. Additionally, it included a comparison of EFs calculated based on HC50_EC50eq_ in this study with 58 PFAS chemicals overlapping with the USEtox database. Furthermore, EFs calculated in this study based on HC20_EC10eq_ were compared with 130 EFs calculated by Saouter et al. (2018) for the overlapping chemicals. The regression analysis with all the experimental data, based on two different EFs calculation methodologies, showed an R^2^ value of 0.94, indicating a very strong correlation. The comparison with the USEtox database resulted in an R^2^ value of 0.83, indicating a strong correlation. However, the comparison with Saouter et al. (2018) yielded an R^2^ value of 0.70, signifying a moderate correlation.

**Table 2.** Overview of the regression analysis between log-transformed effect factors (EFs) [PAF m³ kg^−1^] calculated using two different methodologies, along with available EFs from the USEtox database and Saouter et al. (2018).

Variable (PAF m³ kg⁻¹)	By variable (PAF m³ kg⁻¹)	Correlation (r)	Rsquare (R^2^)	Root mean square error	Covariance	Count	Correlation lower 95%	Correlation upper 95%
log EF (HC20_EC10eq_)	log EF (HC50_EC50eq_)	0.97	0.94	0.35	1.80	367	0.96	0.97
log EF (HC50_EC50eq_)	log EF (HC50_EC50eq_), USEtox	0.91	0.83	0.60	2.18	58	0.85	0.95
log EF (HC20_EC10eq_)	log EF (HC20_EC10eq_), Saouter et al. (2018)	0.84	0.70	0.78	2.10	130	0.78	0.88

*Note*: PAF = Potentially affected fraction of species.

**Table 3.** Overview of the regression analysis between log-transformed characterization factors (CFs) [PAF·m³·d/kg emitted] calculated using two different methodologies (USEtox and adapted USEtox) with effect factors based on both HC50_EC50eq_ and HC20_EC10eq_, with available CFs from the USEtox database and Saouter et al. (2018).

Variable (PAF‧m^3^‧d/kg emitted)	By variable (PAF‧m^3^‧d/kg emitted)	Correlation (r)	Rsquare (R^2^)	Root mean square error	Covariance	Count	Correlation lower 95%	Correlation upper 95%
log CF (HC20_EC10eq_)	log CF (HC20_EC10eq_), adapted	0.73	0.53	1.30	1.91	367	0.67	0.77
log CF (HC50_EC50eq_)	log CF (HC50_EC50eq_), adapted	0.72	0.52	1.30	1.77	367	0.66	0.76
log CF (HC20_EC10eq_)	log CF (HC50_EC50eq_)	0.98	0.96	0.35	3.46	367	0.98	0.99
log CF (HC20_EC10eq_), adapted	log CF (HC50_EC50eq_), adapted	0.97	0.94	0.35	1.79	367	0.96	0.97
log CF (HC50_EC50eq_)	log CF (HC50_EC50eq_), USEtox	0.92	0.85	0.53	2.05	58	0.87	0.95
log CF (HC20_EC10eq_)	log CF (HC20_EC10eq_), Saouter et al. (2018)	0.82	0.68	0.83	2.21	130	0.76	0.87

*Note*: PAF = Potentially affected fraction of species.

### Ecotoxicity characterization factor results

**Figure 3.**
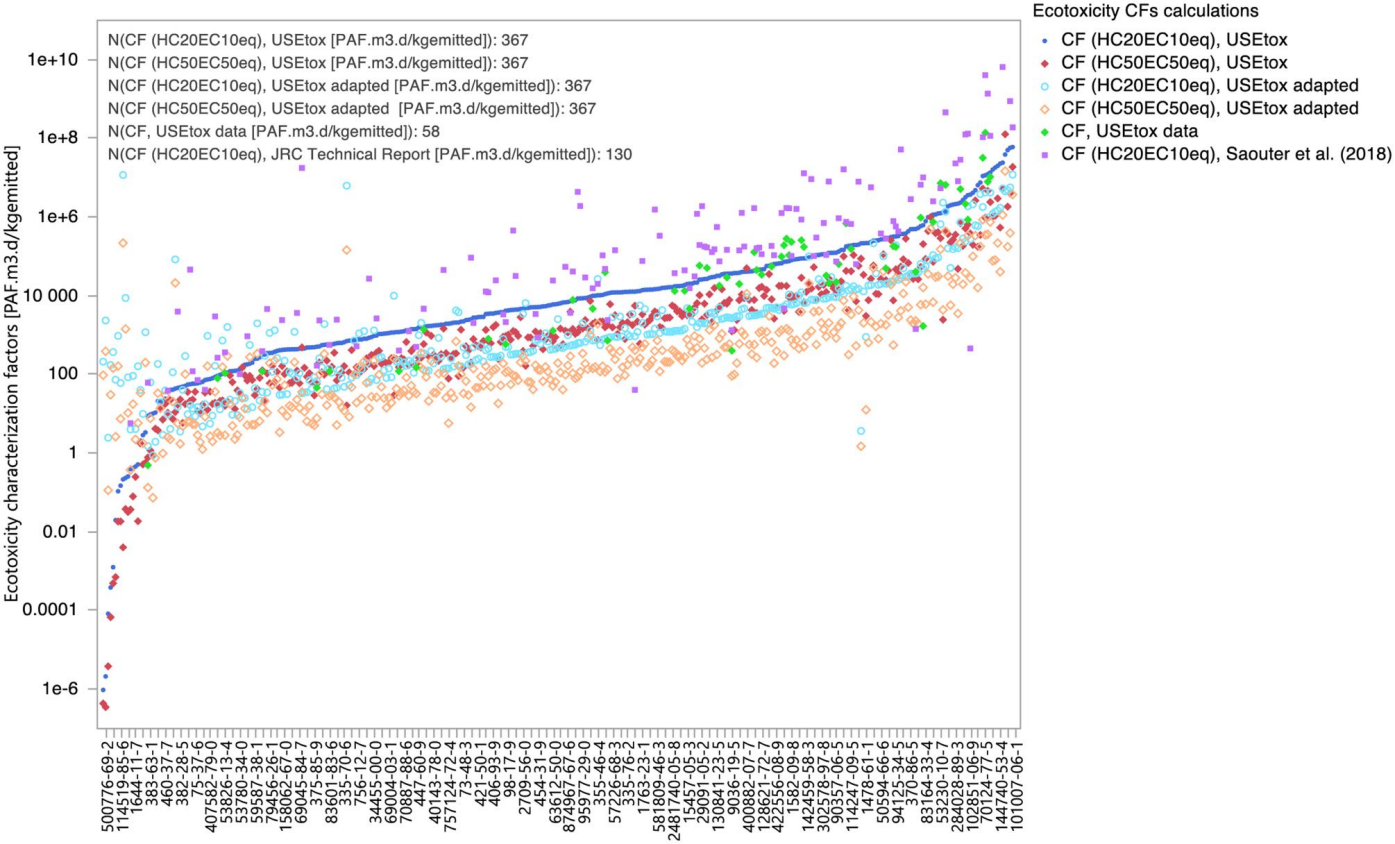
Calculated ecotoxicity characterization factors for 367 PFAS chemicals based on HC20_EC10eq_ and HC50_EC50eq_, including overlaps with the USEtox database (version 2.01) for 58 chemicals (based on HC50_EC50eq_) and overlaps with Saouter et al. (2018) for 130 chemicals (based on HC20_EC10eq_).

In this study, CFs were derived for a total of 367 PFAS chemicals using four methodologies as described in the “Characterization factor calculation tool” section. Further details on the summary statistics of input data and the calculated CFs are available in the online supplementary material Table S2. The calculated CFs exhibit a range of values. To visually illustrate this variation, Figure 3 presents CFs calculated in this study using the four methodologies along with CFs for 58 PFAS chemicals already present in the USEtox database and 130 PFAS chemicals from Saouter et al. (2018). These graphical representations effectively showcase the distribution of CFs, highlighting the range of values.

**Figure 4.**
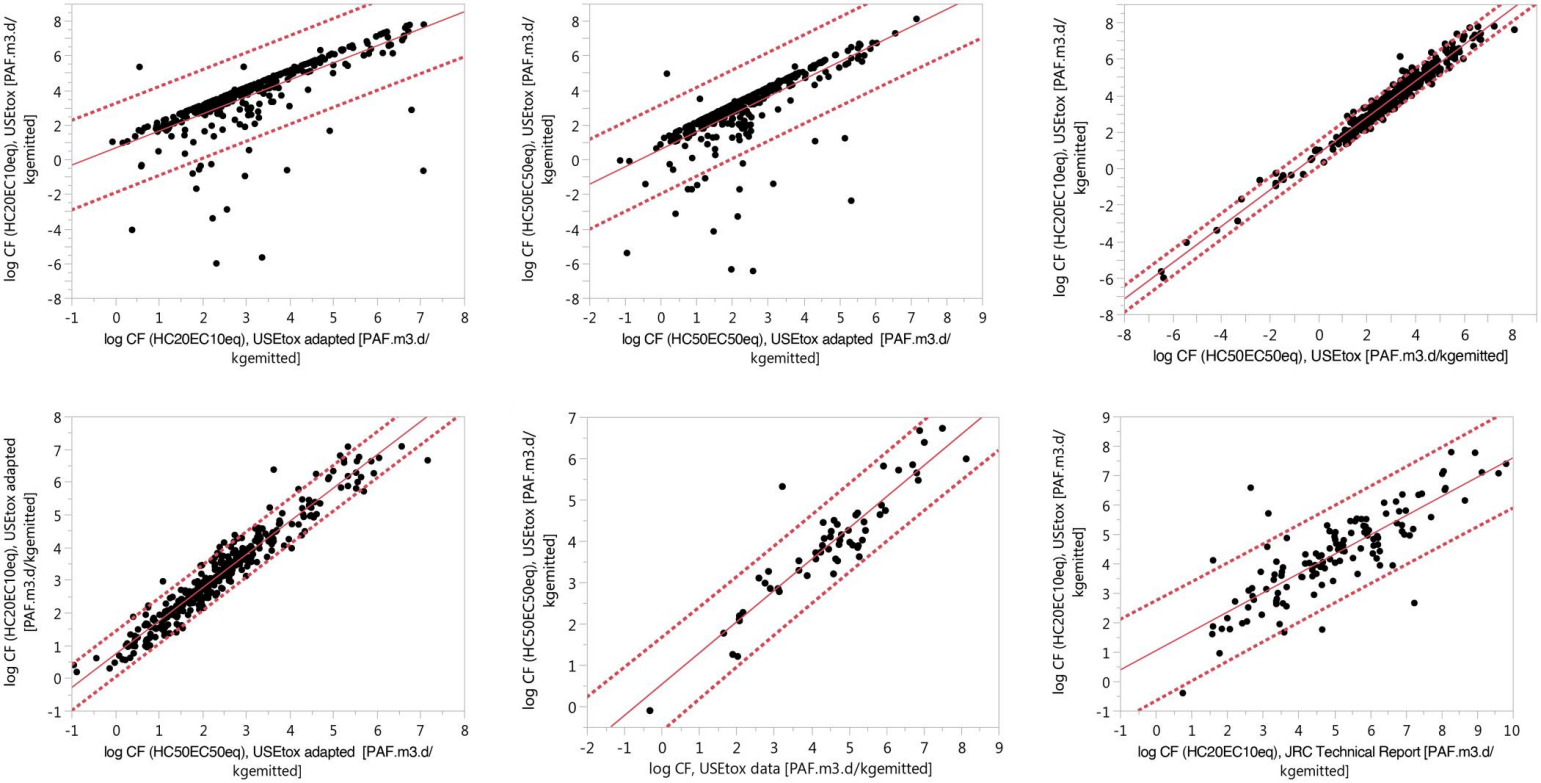
Regression analysis of log-transformed characterization factors (CFs) [Potentially affected fraction of species (PAF)·m³·d/kg emitted] calculated using two different methodologies (USEtox and adapted USEtox) with effect factors (EFs) based on both HC50_EC50eq_ and HC20_EC10eq_, along with available CFs from the USEtox database and Saouter et al. (2018).

It is vital to emphasize that these CFs primarily serve as preliminary indicators, requiring further validation and verification for any substantive applications beyond initial screenings. The disparities in CFs calculated in this study using USEtox and adapted USEtox methodologies primarily stem from variations in XF and FF methodologies, whereas the ecotoxicity data remain consistent. This underscores the influence of these distinct calculation approaches on the calculated CFs.

However, the differences between the USEtox database and Saouter et al. (2018) are based on variations in XF, FF, and EF, as the underlying data are different. For example, the data in USEtox were sourced from the e-toxBase database from the National Institute for Public Health and the Environment (RIVM) by van Zelm et al. (2009; 2007) and the ECOTOX and IUCLID databases as referenced by Payet (2004). These data have been updated with new ecotoxicity information. With changes in data, the CF values also change.

The focus of this study was on the methodology used to calculate CFs based on the USEtox model (version 2.13) and adapted USEtox, whereas Saouter et al. (2018) used the USEtox 2.1 model. Additionally, CFs for an additional 237 PFASs were calculated that were not previously available, using the methodology based on HC20_EC10eq._ Furthermore, 367 CFs were calculated based on the adapted USEtox model, which is specifically suited for PFASs, rather than the general USEtox model (version 2.13) used for all chemicals.

### Comparison of ecotoxicity characterization factors

In this study, a regression analysis was first conducted on CFs calculated by using both the USEtox and adapted USEtox models, based on HC50_EC50eq_ and HC20_EC10eq_, respectively, for 367 PFAS chemicals. Subsequently, comparisons were made between CFs based on HC50_EC50eq_ and HC20_EC10eq_ using only USEtox. Another comparison was made between CFs based on HC50_EC50eq_ and HC20_EC10eq_ using only the adapted USEtox model. Additionally, CFs based on HC50_EC50eq_ calculated in this study were compared with 58 PFAS chemicals overlapping with the USEtox database. Furthermore, CFs calculated in this study based on HC20_EC10eq_ were compared with 130 CFs calculated by Saouter et al. (2018) for the overlapping chemicals. All comparisons focused on CFs are shown in Figure 4 and Table 3.

**Table 4.** Overview of the range of calculated log-transformed EFs [PAF·m³·kg^−1^] and CFs [PAF·m³·d/kg emitted] using USEtox based on both HC50_EC50eq_ and HC20_EC10eq_ within groups with the same number of perfluorinated carbons.

Calculated CFs, USEtox [PAF.m^3^.d/kg emitted]	Count	Number of fluorinated carbons	Mean	*SD*	Minimum	Maximum	Calculated EFs, USEtox [PAF.m^3^.kg^-1^]	Mean	*SD*	Minimum	Maximum
log CF (HC20_EC10eq_)	1	17	−1.70		−1.70	−1.70	log EF (HC20_EC10eq_)	5.13		5.13	5.13
log CF (HC50_EC50eq_)	1	17	−3.15		−3.15	−3.15	log EF (HC50_EC50eq_)	3.68		3.68	3.68
log CF (HC20_EC10eq_)	5	16	−1.01	4.10	−6.02	3.19	log EF (HC20_EC10eq_)	4.09	1.81	2.64	7.24
log CF (HC50_EC50eq_)	5	16	−1.98	3.89	−6.36	2.42	log EF (HC50_EC50eq_)	3.12	1.40	2.29	5.60
log CF (HC20_EC10eq_)	1	15	−0.29		−0.29	−0.29	log EF (HC20_EC10eq_)	5.11		5.11	5.11
log CF (HC50_EC50eq_)	1	15	−1.73		−1.73	−1.73	log EF (HC50_EC50eq_)	3.67		3.67	3.67
log CF (HC20_EC10eq_)	1	13	5.34		5.34	5.34	log EF (HC20_EC10eq_)	4.58		4.58	4.58
log CF (HC50_EC50eq_)	1	13	3.49		3.49	3.49	log EF (HC50_EC50eq_)	2.73		2.73	2.73
log CF (HC20_EC10eq_)	4	12	0.30	2.60	−2.90	2.89	log EF (HC20_EC10eq_)	4.47	2.10	2.87	7.53
log CF (HC50_EC50eq_)	4	12	−0.64	2.61	−3.31	2.03	log EF (HC50_EC50eq_)	3.53	1.55	2.46	5.80
log CF (HC20_EC10eq_)	1	11	3.32		3.32	3.32	log EF (HC20_EC10eq_)	4.07		4.07	4.07
log CF (HC50_EC50eq_)	1	11	1.96		1.96	1.96	log EF (HC50_EC50eq_)	2.71		2.71	2.71
log CF (HC20_EC10eq_)	6	10	2.48	1.42	0.53	4.94	log EF (HC20_EC10eq_)	3.32	0.52	2.66	4.20
log CF (HC50_EC50eq_)	6	10	1.78	1.25	−0.24	3.59	log EF (HC50_EC50eq_)	2.62	0.19	2.33	2.85
log CF (HC20_EC10eq_)	4	9	1.34	3.32	−3.41	4.10	log EF (HC20_EC10eq_)	3.04	0.36	2.67	3.42
log CF (HC50_EC50eq_)	4	9	0.55	3.22	−4.17	2.90	log EF (HC50_EC50eq_)	2.24	0.33	1.92	2.66
log CF (HC20_EC10eq_)	30	8	2.87	1.83	−5.67	4.22	log EF (HC20_EC10eq_)	3.07	0.69	1.13	4.22
log CF (HC50_EC50eq_)	30	8	2.06	1.79	−6.46	3.45	log EF (HC50_EC50eq_)	2.26	0.61	0.37	3.03
log CF (HC20_EC10eq_)	8	7	2.55	1.56	−0.59	4.09	log EF (HC20_EC10eq_)	2.63	0.60	1.33	3.17
log CF (HC50_EC50eq_)	8	7	1.78	1.53	−1.50	3.17	log EF (HC50_EC50eq_)	1.86	0.48	1.17	2.39
log CF (HC20_EC10eq_)	25	6	3.02	1.92	−0.83	7.70	log EF (HC20_EC10eq_)	2.86	1.24	1.27	6.96
log CF (HC50_EC50eq_)	25	6	2.29	1.82	−1.74	6.26	log EF (HC50_EC50eq_)	2.12	1.07	0.53	5.52
log CF (HC20_EC10eq_)	13	5	2.40	1.39	−0.41	4.21	log EF (HC20_EC10eq_)	2.33	0.93	0.74	3.43
log CF (HC50_EC50eq_)	13	5	1.60	1.42	−1.43	3.44	log EF (HC50_EC50eq_)	1.53	0.93	−0.29	2.66
log CF (HC20_EC10eq_)	18	4	3.41	1.15	1.69	6.12	log EF (HC20_EC10eq_)	2.85	1.20	1.13	6.47
log CF (HC50_EC50eq_)	18	4	2.50	0.87	0.77	3.89	log EF (HC50_EC50eq_)	1.94	0.81	0.60	3.74
log CF (HC20_EC10eq_)	8	3	3.19	1.57	1.03	6.57	log EF (HC20_EC10eq_)	2.60	1.55	0.43	5.86
log CF (HC50_EC50eq_)	8	3	2.47	1.42	0.62	5.58	log EF (HC50_EC50eq_)	1.89	1.41	0.02	4.87
log CF (HC20_EC10eq_)	46	2	4.31	1.65	1.01	7.78	log EF (HC20_EC10eq_)	3.72	1.63	0.11	7.22
log CF (HC50_EC50eq_)	46	2	3.64	1.63	−0.07	7.27	log EF (HC50_EC50eq_)	3.05	1.62	−0.97	6.71
log CF (HC20_EC10eq_)	196	1	4.28	1.43	−0.34	7.76	log EF (HC20_EC10eq_)	3.50	1.45	0.40	7.21
log CF (HC50_EC50eq_)	196	1	3.56	1.36	−0.60	8.11	log EF (HC50_EC50eq_)	2.78	1.38	−0.65	7.35

*Note*: EFs = effect factors; PAF = Potentially affected fraction of species; CFs = characterization factors.

The first regression analysis, based on HC20_EC10eq_ CFs using both USEtox and adapted USEtox calculation methodologies, showed an R^2^ value of 0.53, indicating a weak correlation. The second analysis, based on HC50_EC50eq_ CFs using both USEtox and adapted USEtox calculation methodologies, showed an R^2^ value of 0.52, also indicating a weak correlation. The main differences arise because although the EFs are the same, FF and XF are different, leading to different results. This shows that the two methodologies are distinct, with one being general for all chemicals and the other specifically for PFASs. The third comparison, using the same model but different EF calculation methodologies, showed a high correlation, with an R^2^ value of 0.96, indicating that if data are available for EFs with HC50_EC50eq_ based on earlier USEtox calculation methodology, they can be extrapolated to CFs based on HC20_EC10eq_ without recalculating the EFs with HC20_EC10eq_, as they are highly correlated. The same high correlation was found with the adapted USEtox model, showing an R^2^ value of 0.94. The comparison with the USEtox database resulted in an R^2^ value of 0.85, indicating a strong correlation. However, the comparison with Saouter et al. (2018) yielded an R^2^ value of 0.68, signifying a moderate correlation. The differences with the USEtox database and Saouter et al. (2018) calculated CFs are based on variations in XF, FF, and EF, as the underlying data are different.

### Group analysis between CFs and number of perfluorinated carbons

A simplified group analysis was developed to relate the number of perfluorinated carbons with the calculated log-transformed EFs and CFs using the USEtox model based on HC20_EC10eq_ and HC50_EC50eq_, as shown in Table 4. This analysis aimed to determine whether there is any correlation between PFASs with the same number of perfluorinated carbons and their respective EFs and CFs, which could be used to fill data gaps in the absence of the required data to calculate EFs and CFs for PFASs.

**Figure 5.**
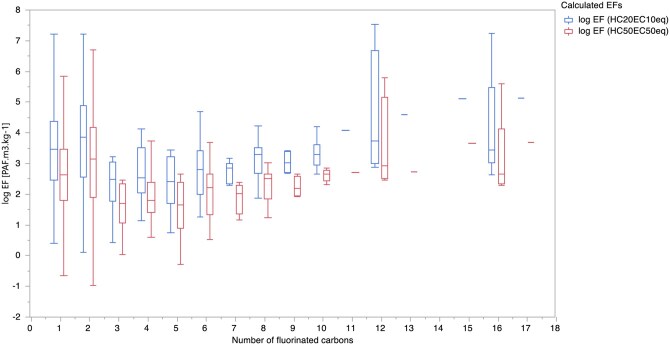
Box plots of calculated log-transformed ecotoxicity factors [Potentially affected fraction of species (PAF)·m³·kg^−1^] derived from experimental ecotoxicity data in the freshwater compartment using USEtox based on both HC50_EC50eq_ and HC20_EC10eq_ corresponding to number of perfluorinated carbons.

**Figure 6.**
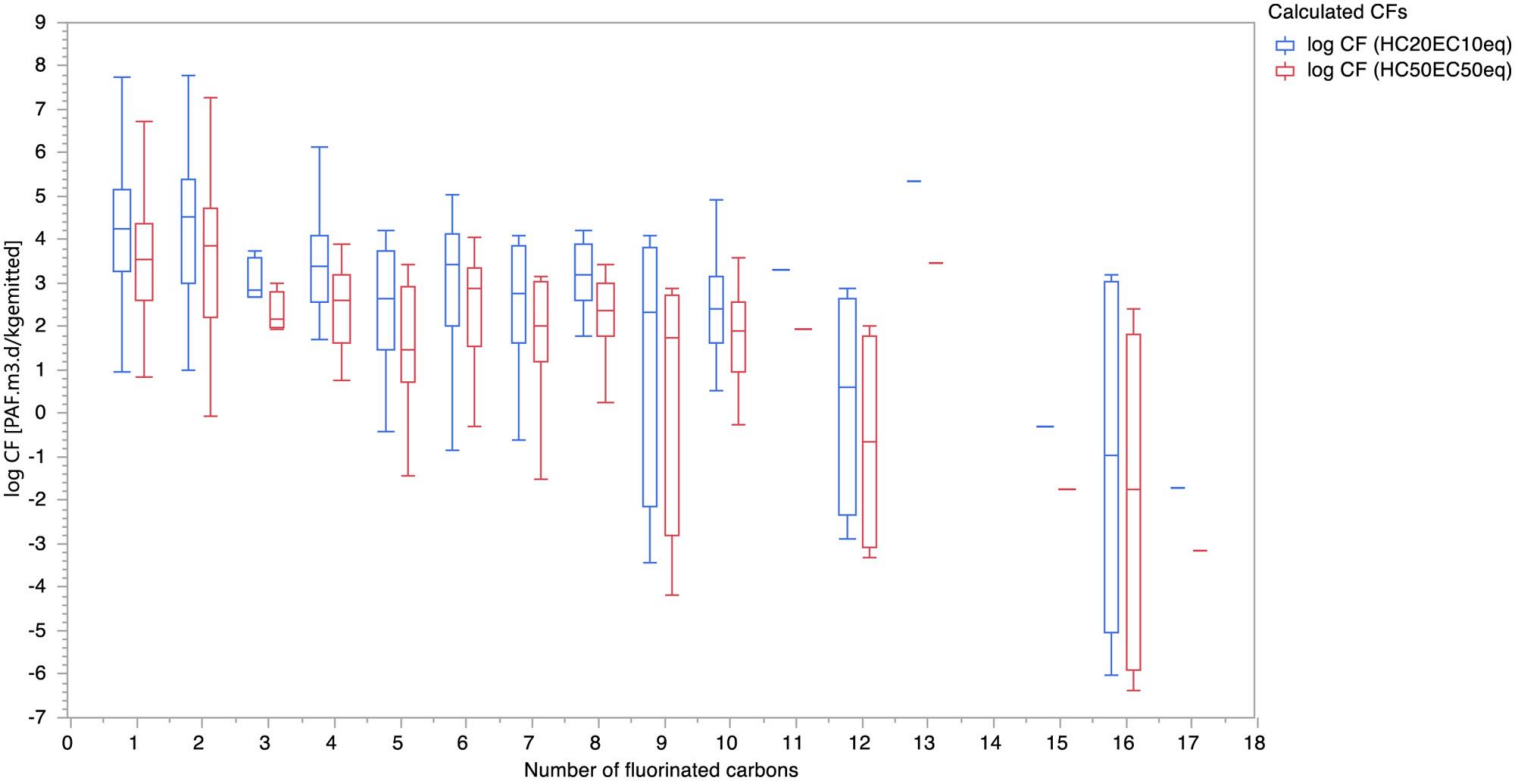
Box plots of calculated log-transformed midpoint characterization factors [Potentially affected fraction of species (PAF).m^3^.d/kg emitted] derived from experimental ecotoxicity data in the freshwater compartment using USEtox based on both HC50_EC50eq_ and HC20_EC10eq_ corresponding to number of perfluorinated carbons.

Table 4 provides the number of fluorinated carbons, the total number of PFASs with that number of fluorinated carbons, and the range of EFs and CFs. The box plots shown in Figure 5 for EFs and Figure 6 for CFs indicate that there is no consistent correlation between an increase in the number of perfluorinated carbons and a corresponding increase or decrease in CFs or EFs. The diversity in CFs for PFAS with the same number of perfluorinated carbons suggests that there is no significant correlation. This result implies that the number of perfluorinated carbons cannot reliably predict the increase or decrease in CFs and EFs, indicating that other factors likely play a more significant role in determining these values.

## Conclusion

PFAS chemicals have raised significant environmental concerns, prompting the need for the inclusion of PFAS ecotoxicity emissions in LCA studies. Characterization factors are crucial for incorporating these chemical emissions into LCA evaluations. Although there are millions of PFAS chemicals, the USEtox database (version 2.01) only covers a few of them. This research addresses this gap by providing experimental aquatic CFs for 367 PFAS chemicals using the USEtox model (version 2.13) and PFAS adapted USEtox, using both HC50_EC50eq_ and HC20_EC10eq_-based calculation methodologies. This effort enriches their representation in LCA evaluations. The study also identified 58 overlapping chemicals with the USEtox organic substances database (version 2.01) and 130 overlapping chemicals with Saouter et al. (2018). This study provided additional CFs for 237 PFASs that were not previously available, using the HC20_EC10eq_-based methodology. Furthermore, the discrepancies observed between USEtox and PFAS-adapted USEtox primarily stem from variations in foundational fate and exposure factor calculation methodologies, whereas the ecotoxicity factors remain consistent. The PFAS group analysis revealed no correlation between the number of perfluorinated carbons and the calculated log-transformed EFs and CFs computed using USEtox models. It is crucial to recognize that PFAS CFs are dynamic and require regular updates both in calculation methodology and available ecotoxicity data to align with the evolving chemical data landscape, ensuring their continued relevance and accuracy.

## Supplementary Material

vjae013_Supplementary_Data

## Data Availability

All data generated during this study are included in this published article and its supplementary information file.
